# Review of Doody's Core Titles in the Health Sciences 2004 (*DCT 2004*)

**DOI:** 10.1186/1742-5581-2-5

**Published:** 2005-06-29

**Authors:** Mark A Spasser

**Affiliations:** 1 Robert B. Greenblatt, MD Library, Medical College of Georgia, 1459 Laney Walker Blvd., Augusta, GA 30912, USA

## Overview

For the last forty years, the *Brandon / Hill Selected Lists *of medical and nursing and allied health books have served as indispensable collection development tools in the medical library community. Following the announcement in April 2004 that the Brandon / Hill Selected Lists would no longer be updated, Doody Enterprises, Inc. decided to develop and publish a list of core titles that would help medical librarians in their collection development decisions. Doody's *Core Titles in the Health Sciences 2004 (DCT 2004) *is the result of the collaborative effort of approximately 200 content specialists, medical library collection development experts, medical book wholesalers, and the publisher's staff.

## History

The "Brandon List," a labor of love for creators Al Brandon and Dorothy Hill, librarians at the Levy Library at Mt. Sinai School of Medicine in New York City, debuted in July 1965 and quickly became vital for medical libraries making collection development decisions. Ultimately split into two lists – one addressing medicine and the other covering allied health and nursing specialties – the Brandon/Hill Lists were updated every two years.

Their initial objective was to develop a list of selected titles for hospital libraries. However, the lists had great reach and influence, and medical libraries of all sizes and types relied on their guidance. Although the librarians had help and support from colleagues, publishers, and medical book wholesalers, they essentially reviewed and made the selections themselves, and they never maintained that their selection process was anything but subjective.

Each new list was published in the **J Med Libr Assoc**, and then the Medical Library Association (MLA) sold reprints of the lists to medical book wholesalers, who, in turn, distributed the lists for free throughout the medical library community. In 2001, the Brandon / Hill Selected Lists became available online () [1,2].

Brandon's death was followed several years later by Hill's retirement, and in April of 2004, the staff of the library at Mt. Sinai announced that they would not be updating the *Brandon / Hill Selected Lists *in medicine, nursing and allied health. The discontinuation of the series left a void in the collection development literature, and the profession was rightly concerned.

## Doody Enterprises as a Solution

At the time publication of the Brandon/Hill Lists ceased, Doody Enterprises already had a solid reputation in publishing collection development tools. Its first venture, a bimonthly print journal called *Doody's Health Sciences Book Review Journal (Doody's Journal) *debuted in 1993, and was conceived as the health sciences equivalent to ALA's journal *Choice*. Developed in close consultation with the company's Library Board of Advisors (LBA), *Doody's Journal *was designed by medical librarians for medical librarians. From 1993 – 1998, the MLA endorsed *Doody's Journal *and its electronic version, *Doody's Electronic Journal*, which first appeared in May, 1995, as a "valuable collection development, cataloging, and reference tool" for its members.

Because of Doody's track record of providing timely and authoritative reviews of newly published books from most of the English-language medical publishers in the world, the company was encouraged to take up the Brandon/Hill mantle. After consultation with their LBA and extensive market research, Doody announced its plans to introduce the inaugural edition of a new Web-based annual publication called *Doody's Core Titles in the Health Sciences *by the 4th quarter of 2004.

## Purpose and Character of the List

The purpose of *DCT 2004 *is to provide a comprehensive, timely, and authoritative list of book and software titles that represent essential knowledge for professionals or students, and that are highly recommended for libraries that serves some segment of the health sciences community. The *DCT 2004*'s scope is comprehensive, with each edition covering titles in 119 specialties in clinical medicine, basic sciences, nursing, allied health and associated health professions (e.g., dentistry, chiropractic, veterinary medicine, history of medicine, medical ethics, etc.).

The Web-based review process allows for timely publication, with availability following the selection process by mere weeks. The list is updated on a weekly basis with new pricing and edition information. Finally, the authoritativeness of the list derives from the individuals involved in selection and the selection process itself.

## The Selection Process

The original list for each of the specialties represented in *DCT 2004 *is selected by Content Specialists, academically-affiliated health sciences faculty who, in most cases, serve (or have served) as Editorial Review Group Chairs for Doody's Book Review Service™. Each list of core book and software titles is then reviewed by a panel of up to three Library Selectors, (collection development medical librarians) who add titles, as they deem necessary, then score each title based on five criteria essential for responsible collection development.

Doody provides both Content Specialists and Library Selectors with Web-based tools that allow access to the titles that appeared in the final issues of the Brandon / Hill Selected Lists; a searchable database of all in-print book and software titles in Doody's Book Review Service™; an aggregated database consisting of all information from Doody's database (including reviews and ratings; tables of contents licensed from Majors; and bibliographic data on all in-print book and software titles from the web sites of the three major medical book distributors.

According to Doody's description of its methodology, "use of the same five scoring tools and criteria for each title and selector – and averaging the scores across multiple selectors – yields a more "objective" measure than simply asking 'is this a core title?"' In addition, the greater the number of selectors scoring titles in a given specialty, the greater the degree of "objectivity" achieved. Doody states that "relying upon scoring by multiple individuals to provide objectivity is a time-tested approach," and is part of their effort to bring evidence-based methodology to collection development.

The five criteria for collection development are: Authoritativeness of Author(s) and Publisher; Scope and Coverage of the Subject Matter; Quality of Content; Usefulness and Purpose; and Value for the Money.

Each title is graded on a scale of 0 – 3. If a librarian feels that for a given criterion a title should not be considered a core title, they assign a score of 0. If a librarian feels that a title does not belong on the list, the selector scores the title a "0" for each of the criteria. For the other scoring options, a "1" means that the librarian judges the book to be "good" in that particular criterion, a "2" translates to "very good," and a score of "3" in any particular criterion means that the library selector judges the title as "excellent" in that aspect. If a library selector cannot grade a title in one or more criteria because of lack of familiarity with the title, the selector gives the title an "NS" (or "not able to score") designation. If a title receives "NS" scores across all criteria from all selectors, its final score reads "Title Not Scored," meaning that the librarian selectors were unable to rate this title based on the collection development criteria and their first-hand knowledge of the title.

## Interpreting the Scores

*DCT 2004 *offers scores for 1,901 titles in 119 specialties. Titles receiving a score of 3.0, which represents 18% of the titles on the *DCT 2004 *list, are visually designated with a symbol and are referred to as "essential core titles." *DCT 2004 *contains 343 "essential core titles". The cost of these titles, based on the retail prices at publication of *DCT 2004 *amounts to $39,292.43.

Titles scoring between 2.6 and 2.9 represent 37% of the titles of the *DCT 2004 *list. The 702 titles falling into this scoring range are designated as "key core titles" and visually represented with a different symbol. These "key core titles" represent an investment of $84,087.47, based on retail list prices as of the time of publication of *DCT 2004*. Estimated costs are calculated based on the current list price of each title on the unique list of titles falling into a given scoring range.

## Notes on How Titles are Listed

Titles are listed by:

Edition In nearly all cases, the latest edition is listed, provided it was available for review. In a few instances, the most recent edition is listed, though not yet available, or a link is provided. This irregularity is due to efforts to publish the inaugural edition in time to be of use for library collection budgets; it should disappear with the next edition of *DCT 2004*.

Volume or Cover Type The goal of the *DCT 2004 *is to offer information about the single volume of a title that is offered in single and multivolume packages; about the hard cover version of a book offered in both hard and soft cover; and to give the most up-to-date retail pricing available.

Publisher Mergers and acquisitions have resulted in core titles changing publishers. Although the *DCT 2004 *strives to list the publisher which currently owns the right to the title, the way titles are listed in the two databases that supplied the information for *DCT 2004 *has made that difficult to do

Scoring A unique attribute of *DCT 2004 *is the score assigned to each title. See the section above for a complete description of the process.

Display options:

When selecting titles, features include **View Mode **(condensed and expanded) and various **Sort By **selections: Author's Last Name, Title, Price, Score, and Copyright Year. Identifying icons are **Essential Core **and **Key Core **titles. Titles can be chosen and viewed in the following ways:

• List Overview and Analysis

• Titles By Specialty

• Unique Title List

• Printable List

## Doody's DCT 2004 – Psychiatry, Pharmacy/Pharmacology, & Nursing Theory

To illustrate both the strengths and weaknesses of the *DCT 2004*, psychiatry from the clinical sciences; pharmacy for the associated health professions and pharmacology from the basic sciences; and nursing theory are used.

In terms of psychiatry, it is clear that most core titles are represented. These include (but are certainly not limited to):

American Psychiatric Association's *Diagnostic and Statistical Manual of Mental Disorders*: Text Revision, 4th Edition, 2000;

Davis's *Neuropsychopharmacology: The Fifth Generation of Progress: An Official Publication of the American College of Neuropsychopharmacology*, 5th Edition, 2002;

Janicak's *Principles and Practice of Psychopharmacotherapy*, 3rd Edition, 2001;

Sadock, Kaplan and Sadock's *Comprehensive Textbook of Psychiatry *– 2 Volume Set, 7th Edition, 2000

Schatzberg's *Manual of Clinical Psychopharmacology*, 4th Edition, 2003, and *The American Psychiatric Publishing Textbook of Psychopharmacology*, 3rd Edition, 2004;

Stahl's *Essential Psychopharmacology: Neuroscientific Basis and Practical Applications*, 2nd Edition, 2000; and

Yudofsky's *The American Psychiatric Publishing Textbook of Neuropsychiatry and Clinical Neurosciences*, 4th Edition, 2002.

In terms of pharmacy from the associated health sciences and pharmacology from the basic sciences, core titles listed include:

ASHP's *AHFS Drug Handbook*, 2nd Edition, 2003;

Allen's *Ansel's pharmaceutical dosage forms and drug delivery systems*, 8^th ^ed., 2005;

*Avery's drug treatment : principles and practice of clinical pharmacology and therapeutics*, 3^rd ^ed., 1987;

Cooper, *The Biochemical Basis of Neuropharmacology*, 8th Edition, 2003;

DiPiro's *Pharmacotherapy: A Pathophysiologic Approach*, 5th Edition, 2002; Evans's *Trease and Evans pharmacognosy*, 15^th ^ed., 2002.

Gahart's *2005 Intravenous Medications: A Handbook for Nurses and Allied Health Professionals*, 2005;

Hardman's *Goodman & Gilman's the pharmacological basis of therapeutics*, 10th ed., 2001;

Katzung's *Basic and Clinical Pharmacology*, 9th Edition, 2004

Gennaro's *Remington's pharmaceutical sciences*, 20^th ^ed.;

Shargel's *Applied biopharmaceutics & pharmacokinetics*, 2005; and

Tietze's *Clinical Skills for Pharmacists: A Patient-Focused Approach*, 2nd ed., 2004.

It is clear that some fundamental texts are missing from the pharmacy/pharmacology lists. For example, Block, Wilson and Gisvold's *Textbook of Organic Medicinal and Pharmaceutical Chemistry*, 11th Edition, 2004; *Goldfrank's toxicologic emergencies*, 7^th ^ed., 2002; and Koda-Kimble's *Applied therapeutics : the clinical use of drugs*, 8^th ^ed. 2005.

With regard to nursing theory, many core titles are listed:

Anderson's *Community as Partner: Theory and Practice in Nursing*, 4th Edition, 2004;

Andrews's *Transcultural Concepts in Nursing Care*, 3rd Edition, 1999;

Cherry's *Contemporary Nursing: Issues, Trends and Management*, 2nd Edition, 2002;

Chinn's *Integrated Knowledge Development in Nursing*, 6th Edition, 2004;

Chitty's *Professional Nursing: Concepts and Challenges*, 4th edition, 2004;

Fawcett's *Relationship of Theory and Research*, 3rd edition, 1999;

Kim's *Nursing Theories: Conceptual and Philosophical Foundations*, 1999;

Orem's *Nursing: Concepts of Practice*, 6th Edition, 2001; and

Tomey's *Nursing Theorists and Their Work*, 5th Edition, 2002.

Missing, however, are: Fawcett's *Contemporary nursing knowledge :analysis and evaluation of nursing models and theories*, 2005; and some version of *Nightingale's Notes on Nursing*.

Thus, the strengths and weaknesses of Doody's *DCT 2004 *are illustrated by an analysis of the selection and rating of items in the psychiatry, pharmacology, and pharmacy sections. In the three disciplines discussed above, the *DCT 2004 *is more or less complete, but the rating system has too little variation (1–3) to measure meaningful differences between items. For instance, what is the true difference between mean scores of 2.5 and 2.6? or between 2.9 and 3.0? This overly restrictive range results in a system as necessarily idiosyncratic as the Brandon-Hill lists it *de facto *replaces. Take, for instance, the ratings of such foundational texts as the American Psychiatric Association's *Diagnostic and Statistical Manual of Mental Disorders *or Sadock, Kaplan and Sadock's *Comprehensive Textbook of Psychiatry*: the former is rated 3.0 and the latter 2.9. It is hard to understand how these classics are not rated the same. Not to put to fine a point on it, but if 3.0 is the highest rating of the most important psychiatric texts (the two Essential Core texts are each given a 3.0), while the mean score for Key Core titles is about 2.75, then is this mean difference between "Essential" and "Key" a distinction without a difference?

Restriction of range problem could result in spurious relationships or the attenuation of significant relationships; that is, a scale with restricted range is simply less sensitive to meaningful differences [3,4]. For example, when the titles are distributed by score, we see that 189 titles are scored at 2.9, 158 at 2.8, 206 at 2.7, and 183 and 2.6. It's difficult, if not impossible, to decipher how these numbers are representing quality differences. As with many poorly explained phenomena modeled as bell curves, most items fall into the upper middle quartile.

Moreover, the owners and designers of *DCT 2004 *have a different interpretation of "objectivity" than what it commonly refers to, at least in terms of scale construction. For example, as mentioned earlier in the review, they state that "Relying upon scoring by multiple individuals to provide objectivity is a time-tested approach." In terms of psychometric test construction, this refers to reliability not to objectivity. Again from earlier in the review, "Use of the same five scoring tools and criteria for each title and selector – and averaging the scores across multiple selectors – yields a more objective measure than simply asking 'is this a core title."' This is typically a method to establish a scale's construct validity, not its objectivity. In fact, I think it's safe to say that objectivity is not considered a scale property.

Price seems to have little or no association with rating. The average cost of an Essential Core title is $114.56 and $119.78 for a Key Core title. The selection of key texts seems to be adequate, at least for the four disciplines discussed in this review. For nursing, Doody's *DCT 2004 *is certainly not as comprehensive as the Brandon-Hill in Nursing (for example, the 2002 edition of the *Print Nursing Books and Journals 2002 *contains 370 nursing books), but given its far more complex selection and elaborate rating systems, it may strive for comprehensiveness, but achieve selectivity. Perhaps, a way to resolve this seeming paradox is to acknowledge that while the *DCT 2004 *is inclusively comprehensive by covering most of the health sciences, it is more selective in terms of what comprises any given discipline.

## Conclusion

While the construction of the *DCT 2004 *is founded on some shaky methods and assumptions, it is still a valuable and welcome addition collection development tool. As a tool for health sciences collection development, the *DCT 2004 *lists and rates the preponderance of important texts in the basic, clinical, and associated health sciences.

While not entirely successful, the developers of the list have given its conception and realization very careful consideration, and while not more objective than the Brandon Hill it purports to replace, the *DCT 2004 *is certainly more thoughtful in terms of it's selection and rating criteria. Notwithstanding its faults, the *DCT 2004 *is an important resource for health sciences librarians who are responsible for developing and maintaining monographic collections.

## List of Abbreviations Used

LBA – Library Board of Advisors

*DCT 2004 *– Doody's Core Titles

## Competing interests

I was a Library Selector for

• Radiologic Technology

• Respiratory Therapy

• Case Management

• Research

• Theory

in the ***DCT 2004*.**
